# Exploring the therapeutic potential of scorpion venom to mitigate colorectal cancer biomarkers in rats

**DOI:** 10.1186/s43046-025-00311-0

**Published:** 2025-08-28

**Authors:** Wesam M. Salama, Sara O. Radwan, Elsayed I. Salim

**Affiliations:** Tanta University, Tanta, Egypt

**Keywords:** Aberrant crypt foci, Cell cycle arrest, Colorectal cancer, 1,2-Dimethylhydrazine, Scorpion venom, Mucin-depleted foci

## Abstract

Colorectal cancer (CRC) is the second leading cause of cancer-related mortality worldwide. The limitations of conventional therapies, namely severe side effects and the emergence of drug resistance, underscore the urgent need for novel and more effective treatment strategies. Natural products, including bioactive compounds derived from scorpion venom (SV), have demonstrated promising anticancer properties in various studies. This study aimed to investigate the potential chemopreventive and therapeutic effects of *Leiurus quinquestriatus* venom (LQV) and *Androctonus bicolor* venom (ABV) against chemically induced CRC in a rat model. Male rats were randomly assigned to four groups: Group 1 (Gp1) (control), Gp2 (CRC induced using 40 mg/kg 1,2-dimethylhydrazine (DMH), administered subcutaneously for 4 weeks), and Gp3 and 4 (DMH-induced CRC treated intraperitoneally with 0.025 mg/kg LQV and 0.05 mg/kg ABV, respectively, for 11 weeks). At the end of the experimental period, colon tissues were collected for histopathological examination, tumor biomarker analysis, gene expression profiling, cell cycle distribution, and apoptotic assays. Both LQV and ABV significantly reduced the number of aberrant crypt foci (ACF) and mucin-depleted foci (MDF) while enhancing the number of goblet cells in colonic mucosa. Treatment also resulted in a marked downregulation of proliferating cell nuclear antigen (PCNA) and cyclin D1 and upregulation of the tumor suppressor gene PTEN. Moreover, flow cytometry analysis revealed an increase in late apoptotic cells and cell cycle arrest at sub-G1 and G0 phases in venom-treated groups. These findings suggest that LQV and ABV possess notable anti-CRC activity through modulation of proliferation, apoptosis, and gene regulation, highlighting their potential as candidates for alternative CRC therapies.

## Introduction 

Colorectal cancer (CRC) is the third most common type of carcinoma, impacting approximately 1 in 25 women and 1 in 23 men. It is the second leading cause of cancer-related deaths, responsible for over 608,000 fatalities worldwide and accounting for 8% of all cancer-related mortalities [1]. Surgical excision is the main treatment for resectable CRC, while immunotherapy, chemotherapy, and radiation therapy are common treatments for unresectable CRC. These treatments do have some disadvantages, including cytotoxicity to healthy cells and a lack of specificity, which might result in secondary complications [2]. These therapies may be used in combination, depending on the extent and progression of colorectal cancer. However, almost 50% of patients relapse with acquired multidrug-resistant colorectal cancer, even after combination therapy [3].

CRC biomarkers, specifically aberrant crypt foci (ACF)—an early alteration in CRC neoplasia—and mucin-depleted foci (MDF), which are precancerous lesions observed in high-risk individuals exposed to carcinogens, serve as important indicators of neoplastic changes in the colonic mucosa [4]. Oxidative stress plays a crucial role in the initiation and progression of colorectal cancer (CRC), primarily through the accumulation of reactive oxygen species (ROS) that damage DNA, proteins, and lipids. The body’s defense against oxidative stress relies on antioxidant enzymes such as superoxide dismutase (SOD), catalase (CAT), glutathione peroxidase (GPx), and glutathione S-transferase (GST), which neutralize harmful free radicals and maintain redox balance. When these enzymes are overwhelmed or impaired, oxidative damage increases, promoting carcinogenesis. Malondialdehyde (MDA), a product of lipid peroxidation, is commonly used as a biomarker of oxidative stress and is often elevated in CRC. Therefore, assessing the activity of antioxidant enzymes and levels of oxidative markers provides critical insight into the redox status during colorectal tumor development and the efficacy of potential chemopreventive agents [5]. Conventional treatments for CRC, such as surgery and chemotherapy, can cause DNA damage or activate several signaling cascades—such as cell cycle arrest, inhibition of global translation, and DNA repair—which may ultimately lead to cancer cell death [6]. However, as numerous studies have shown, including those in molecular pathogenesis and epidemiology, the effectiveness of chemotherapy varies depending on the cancer subtype. The primary challenges related to chemotherapy are cytotoxicity, drug resistance, and adverse effects.

Various complex natural products from plant and animal sources have produced a large number of useful compounds for the development of novel medications. Numerous natural compounds have demonstrated strong biological activity against bacteria, cancer, and inflammation [7–10].

There are many different kinds of scorpions. The most lethal scorpion species for humans belong to the Buthidae family, which includes *Leiurus quinquestriatus* and *Androctonus bicolor* [11]. Components of scorpion venom (SV) have been investigated for medicinal purposes, and various pharmaceutical applications have been documented [10]. SV—composed of amino acids, nucleotides, oligopeptides, and other water-soluble compounds—is a promising resource for the development of innovative drugs due to its diverse biological properties. Recent studies have shown that the venom induces apoptosis, releases cytochrome C, and causes cell cycle arrest [10, 12]. It may also serve as a valuable component for the development of new drugs with antileishmanial, antimalarial, antitoxocariasis, antidiabetic, and anti-arthritic properties [13–15]. Interestingly, SV peptides have demonstrated potent cytotoxic, antiproliferative, and apoptogenic effects against various cancer cell lines [16]. The ability of SV to induce necrosis or apoptosis in cancer cells may underlie its cytotoxicity [17]. For instance, venom from *Odontobuthus doriae* caused necrosis, apoptosis, and DNA fragmentation and reduced viability in human neuroblastoma cells [18]. Additionally, a prior study found that the venom of *Androctonus amoreuxi* reduced tumor size and volume and downregulated VEGF expression in Ehrlich solid tumors [19].

However, the effectiveness of *L. quinquestriatus* venom (LQV) and *A. bicolor* venom (ABV) in chemically induced CRC in rats has not yet been investigated. Therefore, the current study aimed to evaluate the anti-tumor effects of LQV and ABV on chemically induced CRC in a rat model, as well as to assess their antioxidant properties in liver and colon tissues. The study also examined their effects on histopathology, immunohistochemistry, gene expression, apoptosis, and cell cycle regulation in the colonic tissues of CRC-induced groups.

## Materials and methods

### Collecting of scorpion and venom preparation

In August 2022, 100 scorpions were caught by proficient hunters from Aswan, Upper Egypt, and the Mediterranean northern coast, Egypt. The collected samples were transferred to the invertebrate lab, Zoology Department, Faculty of Science, Tanta University, Egypt, in plastic, well-aerated containers. The samples were identified by an expert in the invertebrate lab. After identification, *L. quinquestriatus* and *A. bicolor* scorpions were separated into two containers and were milked using electrical stimulation at 12–17 V [20]. The venom was centrifuged, lyophilized, and kept at − 20 °C before being used. Different concentrations from *L. quingestriatus* venom (LQV) and *A. bicolor* venom (ABV) were prepared from the lyophilized venom. Sublethal doses were prepared according to [9].

### Experimental animals

Fifty-five male Sprague–Dawley rats, weighted 170 ± 20 g, were obtained from VACSERA, Giza, Egypt. Rats were housed at 23 ± 2 °C and 55 ± 5% relative humidity after a week of living in the circumstances of the animal facility. Animals were observed every day for their body weights, food and drink consumption, and general health.

### Ethical statement

The animals were treated according to the ethical standards with the ARRIVE guidelines and by the National Institute of Health Guide for the Care and Use of Laboratory Animals (NIH Publications No. 8023, revised 1978). The Tanta University’s Faculty of Science’s Animal Care and Use Committee approved the experimental protocol (no.: IACUC/SCI-TU-0270).

### Experiment design

**Fig. 1:**
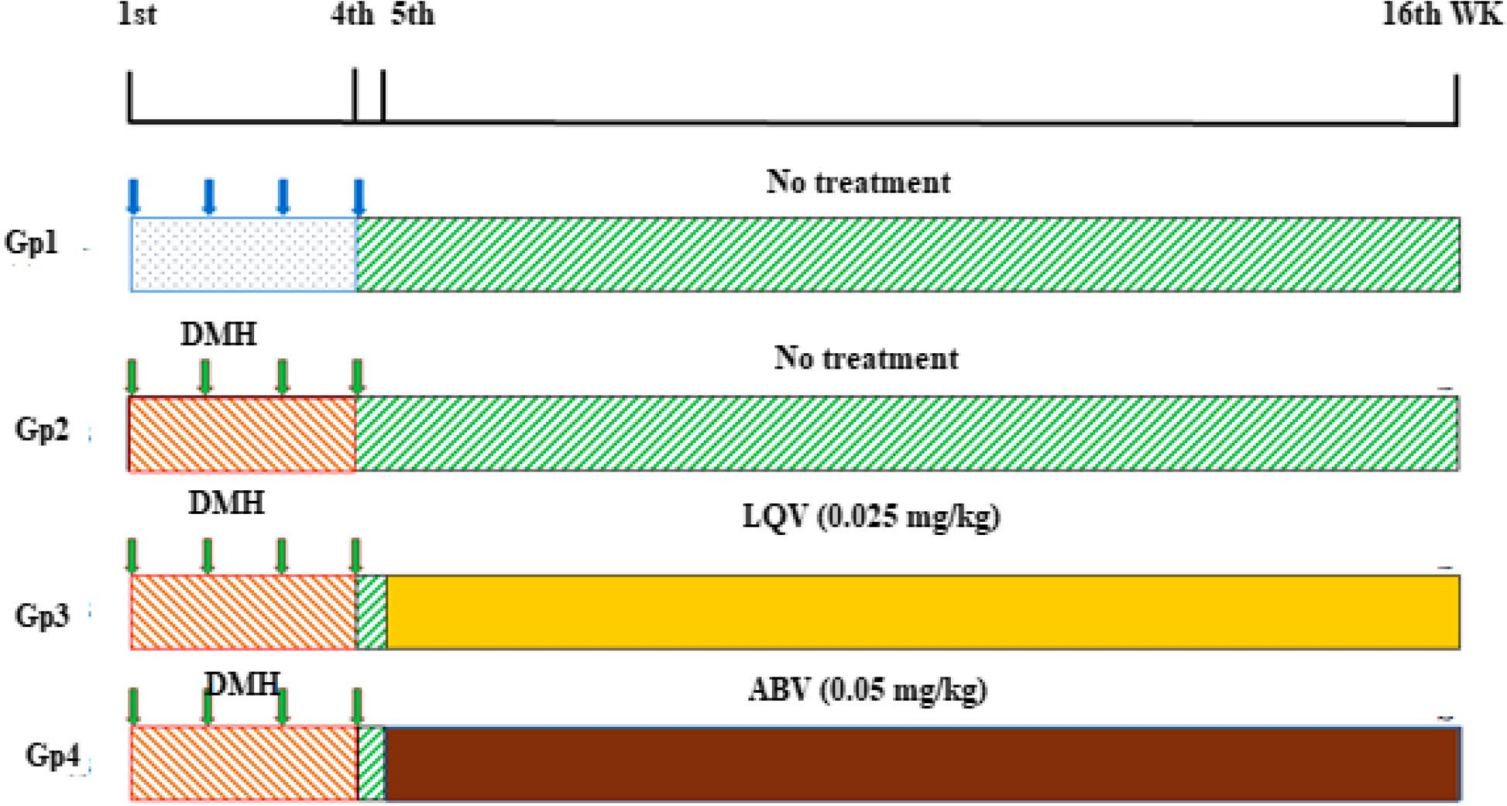
The experimental design of CRC chemically induced rat groups. Gp1, control group; Gp2, CRC-induced group by DMH; Gp3, DMH/LQV treated; Gp4, DMH/ABV treated; *DMH*, carcinogen 1,2-dimethylhydrazine; LQV, *L. quinquestriatus*, venom; ABV, *A. bicolor* venom

Sprague–Dawley male rats were randomly divided into four groups (Fig. 1). The first group (Gp1) (*n* = 10) served as the control group that was injected subcutaneously (s.c) with 0.9% saline. The second group (*n* = 15) was s.c. injected with 1,2-Dimethylhydrazine (DMH) (40 mg/kg b.wt) for 4 weeks (wk) for CRC induction [21]. Gp3 was injected intraperitoneally (i.p.) with 1/20 *LD*_50_ of LQV (0.025 mg/kg) for 11 weeks (from week 5 to week 16) [14]. Gp4 was treated with 1/20 *LD*_50_ of ABV (0.05 mg/kg) via the same route and for the same period as Gp3. At week 16, the end of the experiment, the animals were anesthetized using 3% isoflurane and sacrificed. The rats were dissected, and the liver and colon were separated for further analysis.

Utilizing our most recent study (unpublished data), we investigated the potential impact on colorectal cancer of 1/20 of the sublethal doses of LQV and ABV. Using the *LD*_50_ doses of LQV and ABV, these results were obtained in male Sprague–Dawley (SD) rats.

### Tissue preparation

Liver tissue was perfused with phosphate-buffered saline (PBS) during necropsy to eliminate red blood cells and clots. The liver was cut into tiny pieces to prepare a homogenate. Colons were removed, inflated with 0.09% saline, cut longitudinally, and flushed with saline. Colons from 10 rats were kept in 10% phosphate-buffered formalin for histopathological and immunohistochemistry examinations. Colons from the remaining five rats from each CRC chemically induced group had their colon mucosa scraped out using sterile glass slides, which were then kept at − 80 °C for biochemical and molecular analysis [21].

### Determination of the antioxidant enzymes/oxidant biomarkers in rat liver and colon tissues

The liver and colon homogenates were centrifuged, and the supernatant was assayed using commercial kits (Biodiagnostic, Egypt) for malondialdehyde (MDA), glutathione peroxidase (GPX), superoxide dismutase (SOD), catalase (CAT), and glutathione S-transferases (GST) analysis, in accordance with the protocols described by [22–26], respectively. Briefly, for MDA, mix TBA (thiobarbituric acid + acetic acid + SDS) reagent with the supernatant from liver and colon homogenates, heat at 95 °C for 30–60 min, and read absorbance at 532 nm. GPX activity was measured by evaluating the decrease in NADPH absorbance at 340 nm. Xanthine-xanthine oxidase was used for SOD activity evaluation; the reduction of nitroblue tetrazolium at 560 nm was measured. CAT activity was measured by mixing samples with H_2_O_2_ substrate and reading absorbance at 240 nm. Use 1-chloro-2,4-dinitrobenzene as substrate with reduced glutathione (GSH) to detect GST activity and then measure the formation of GSH-CDNB conjugate at 340 nm.

### Histopathological examinations of colon in CRC-induced groups aberrant crypt foci and mucin-depleted foci (MDA) counting

In histopathological examination, aberrant crypt foci (ACF) counted according to the method of [21]. Briefly, the colon was inflated with cold saline, opened longitudinally, and rinsed in saline. The colon was separated into three parts: proximal, middle, and distal with measurements of their length and width. The separated parts were fixed in 10% phosphate-buffered formalin for at least 48 h and were stained with 0.1% methylene blue for 3 min. ACF was examined under a light microscope, and the numbers were counted and divided by the total number of aberrant crypts to evaluate the crypt ACF that showed variations in its multiplicity, such as foci containing 1 crypt (1AC), foci with 2 or 3 crypts (2ACs and 3ACs), or larger foci with 4 or more crypts (≥ 4ACs), which were counted and separated into categories. Images were shot, and then the samples were stored again in buffered formalin.

For mucin-depleted foci (MDF) counting, colons were re-stained with Alcian blue-neutral red (AB-NR) according to [27]. The colonic epithelial normal mucosa appeared as a reddish background (NR staining) dotted with blue spots representing the opening of normal crypts full of mucus stained with AB. The MDF was distinguished from this blue-dotted background as a reddish spot in which the crypts do not produce mucin.

### Mucin histochemical investigations of colon in CRC-induced groups

Colon tissue samples were fixed in a 10% neutral buffered formalin solution and then processed to create paraffin slices that were 4-μm thick. One method for examining structures with high concentrations of carbohydrate molecules is to use the periodic acid– Schiff reaction stain (PAS). When the staining process is finished, the glycogen, glycoprotein, glycolipids, and mucins all turn red or magenta. Aldehydes are produced when the hydroxyl groups of nearby sugar molecules are oxidized by the highly oxidized iodine known as periodic acid. Following this stage, the aldehyde and Schiff reagent react, giving the aldehyde a magenta hue that can be seen [28].

### Immunohistochemistry investigation and evaluation of the labeling index (LI)

For immunohistochemistry investigations (IHC), 4-µm colon tissue sections were microwaved in sodium citrate buffer, blocked for endogenous peroxide activity, and incubated in 3% H₂O₂ in methanol. To prevent nonspecific binding, slides were treated with Ultra-Block (UltraVision Plus Detection System, Thermo Scientific, USA) and then washed in tris-buffered saline (TBS). They were incubated overnight at 4 °C with a 1:1000 dilution of proliferating cell nuclear antigen (PCNA) rabbit polyclonal antibody (SAB2108448, Sigma Aldrich). After TBS washing, sections were raised in the Thermo Scientific Biotinylated Goat Anti-Polyvalent system, further incubated with streptavidin peroxidase, and developed using a 3′-diaminobenzidine (DAB) solution. Counterstaining was done with hematoxylin. Positively stained cells showed reddish-brown nuclear staining, while negatively stained cells appeared blue. Immunohistochemistry was performed on 2 out of every 3 colon sections, totaling at least 10 sections from 10 animals. The labeling index (LI%) was calculated by dividing the total number of positively stained nuclei by the total number of nuclei per crypt and multiplying by 100, using data from 15 to 20 high-power fields per section [29].

### Gene expression of PTEN and cylin-D1 in colonic tissue of CRC-induced groups using quantitative real-time PCR

The total RNA was extracted from colonic tissues using commercial kits (QIAGEN, Analytik Jena Biometra AG, Berlin, Germany) and quantified using a NanoDrop 2000 (Thermo Scientific, San Jose, CA, USA). The reverse transcriptase kit (QIAGEN, Germany) was then used to reverse transcribe the RNA into cDNA. For RT-PCR, the following primers were utilized: *PTEN*: forward, 5′-GGAAAGGACGGACTGGTGTA-3′, and reverse, 5′-TGCCACTGGTCTGTAATCCA-3′; *cyclin D1*: forward, 5′-ATGCTAGAGGTCTGCGAGGA-3′, and reverse, 5′-GGCTCCAGAGACAAGAAACG-3′; and *β-actin*: forward, 5′-AAGATCCTGACCGAGCGTGG-3′, and reverse, 5′-CAGCACTGTGTTGGCATAGAGG-3′. Applying the Applied Biosystems Steps One™ instrument and the SYBR GREEN PCR Master Mix (QuantiTect® SYBR® Green PCR for quantitative, real-time PCR and two-step RT-PCR utilizing SYBR Green I kit), qRT-PCR was performed in two steps. The comparative CT approach, which is based on the ratio of the target genes’ RNA amount to that of the reference gene (B-actin), was used to calculate the amount of mRNA.

### Determination of apoptosis and cell cycle of tumor cells in colon of CRC-induced and -treated rat groups

DMH-, LQV-, and ABV-treated rats were used to collect tumor cells from their colons, which were subsequently resuspended in PBS at a density of 1 × 106 cells/mL. Annexin-V and PI (5 μL/each) were combined with 100 µl (100 μL) of tumor cell suspension, and the mixture was then incubated for 15 min at 25 °C in the dark. After that, 400 μL of PBS was added. A BD FACSCanto II flow cytometer was used to aspirate and examine the labeled cells; at least 10,000 events were collected per sample. Each sample was analyzed in triplicate.

For analysis of the cell cycle, tumor cells from the colon of CRC-induced and SV-treated groups were fixed overnight in a cold ethanol (70%) at 4 °C. Then, the cell pellets were washed, centrifuged, and resuspended in PI/RNase staining buffer. The final product of cells was analyzed, and the cell-cycle stage was determined using CellQuest software (Becton Dickinson, San Jose, CA, USA) [30]. The results were expressed as percentages of cells in different phases (G0/G1, S, G2/M) or apoptotic stages (early, late). *Statistical analysis* was performed using one-way ANOVA followed by Tukey’s post hoc test. A *p*-value < 0.05 was considered statistically significant.

### Statistical analysis

Using the Statistical Package for Social Science ver. 17 (USA), the chi-squared test was used to analyze percentage data, and the *t*-test or analysis of variance was used to evaluate group values represented as means ± standard deviations. A statistically significant value was defined as *p* < 0.05.

## Results 

### The oxidative biomarker levels and antioxidant enzyme activities in the liver and colon of CRC-induced and -treated groups

**Fig. 2:**
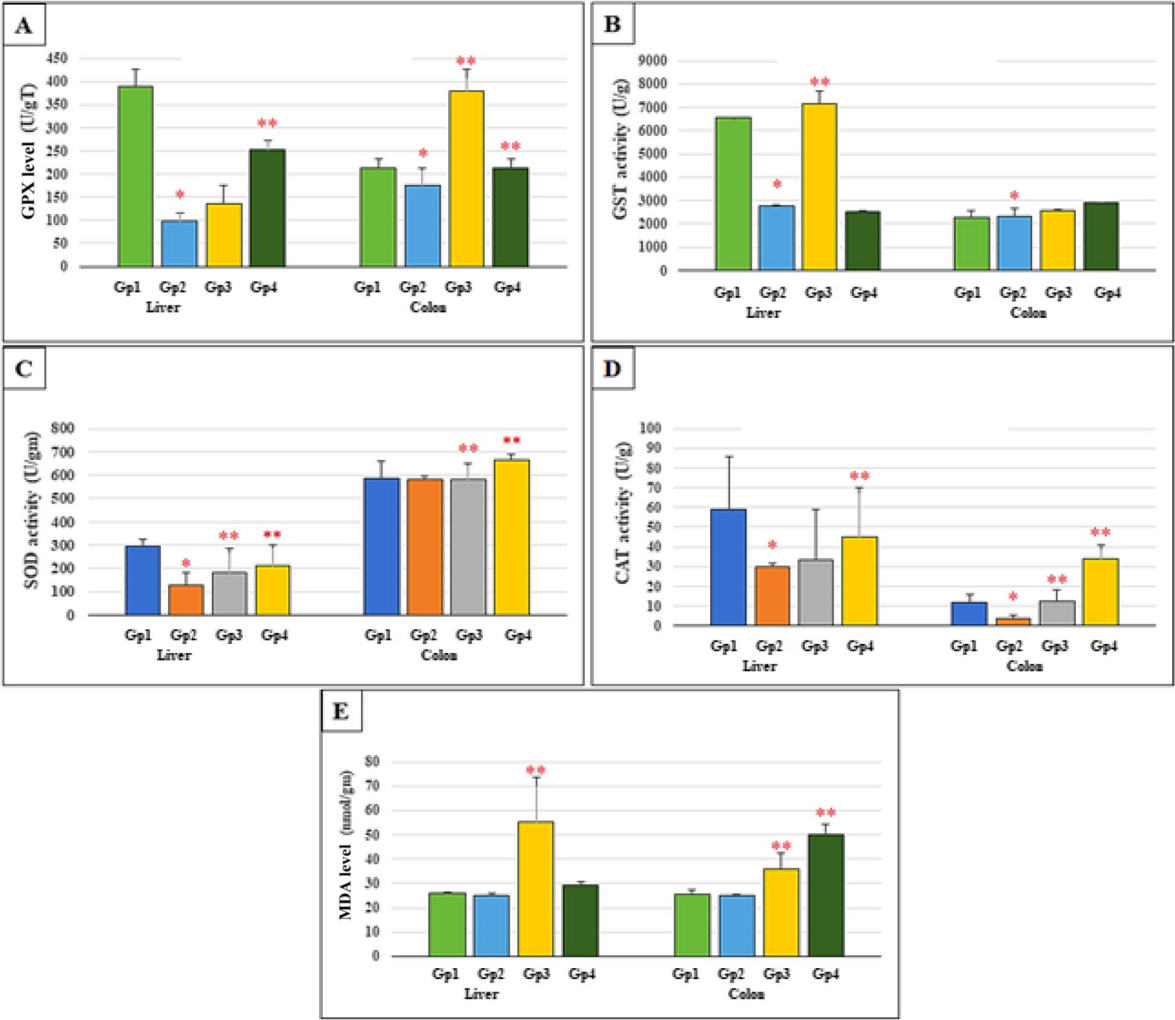
The oxidative biomarker levels and antioxidant enzyme activities in the liver and colon of CRC-induced and -treated groups. Gp1, normal control; Gp2, *L. quinquestriatus* venom injected; Gp3, *A. bicolor* venom injected. *Significant vs. Gp1 *p* < *0.05*. **Significant vs. Gp2 *p* < *0.05*. **A** GPX, cellular glutathione peroxidase. **B** GST, glutathione S-transferase. **C** CAT, catalase. **D** SOD, superoxide dismutase. **E** MDA, malondialdehyde. *t*-test analysis

The levels of cellular glutathione peroxidase (GPX), glutathione S-transferase (GST), and malondialdehyde concentration (MDA),and the activities of superoxide dismutase (SOD) and catalase (CAT) in the liver and colon were evaluated (Fig. 2). In liver tissues, the DMH group exhibited lower activity levels of all antioxidant enzymes than the normal group. Comparing the DMH/ABV-treated group to DMH/LQV-treated group and DMH-only treated group, there was a considerable increase in GPX activity in the DMH/ABV-treated group. In contrast to the other groups, control, DMH, and the DMH/LQV group, GST activity was substantially greater in the DMH/LQV group, while it was slightly lower in the DMH/ABV than in the DMH groups. Compared to the DMH and DMH/LQV groups, there was a rise in SOD activity, but the DMH/ABV-treated group showed a greater increase. Similarly, the DMH/LQV group showed a rise in CAT activity, whereas the DMH/ABV group showed a greater increase. As demonstrated, MDA activity increased dramatically in the DMH/LQV and increased in the DMH/ABV group in comparison to the DMH-only treated group.

In the colon mucosa, it was observed that DMH-treated group had lower GPX activity than control group, that DMH-treated group had significantly lower CAT activity than control, that DMH-treated group had somewhat higher GST activity than control, and that DMH-treated group had slightly lower SOD activity than control group. On the other hand, MDA activity did not alter between DMH-treated and control groups. GPX activity in DMH/LQV-treated group was significantly higher than that of all other groups treated with ABV and DMH only as well as the control group. While in DMH/ABV treated group, GPX activity was higher than in DMH treated group GST activity increased insignificantly in DMH/LQV-treated group and in DMH/ABV-treated group when compared to DMH-treated group. SOD and CAT activities were significantly increased in DMH/LQV-treated group and also in DMH/LQV-treated group compared to DMH-treated group. On the other hand, MDA level increased dramatically in DMH/ABV and DMH/LQV over DMH-treated groups.

### The treatment with LQV and ABV decreased the ACF number in colon in CRC-induced groups

**Fig. 3:**
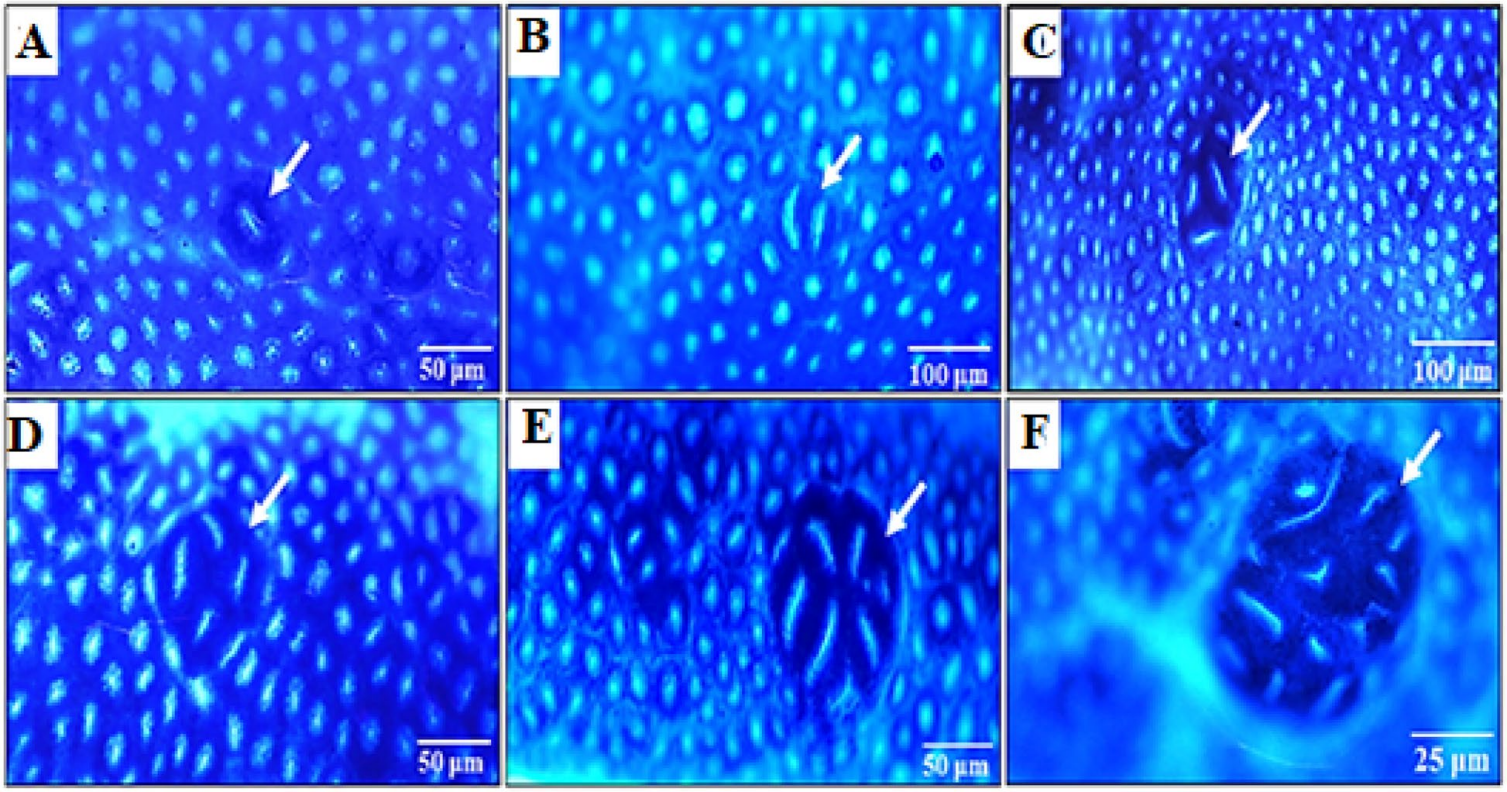
Photomicrographs of colons stained with methylene blue. **A** Foci with one aberrant crypt (one AC). **B** Foci with two ACs. **C** Foci with three ACs. **D** Foci with four ACs. **E** and **F** Larger foci with ≥ 4 ACs

The total number of ACF and the numbers for the various crypt criteria are displayed in Table 1 and Fig. 3. A total of 100% of the animals receiving DMH treatment had an incidence of ACF. Rats treated with LQV and ABV groups had considerably lower total amounts of ACF (*p* < 0.05) than the DMH-only group. The tiny ACF with one, two, or three aberrant crypts (ACs) and large foci with ≥ 4 aberrant crypts (≥ 4 ACs), which are more prone to develop into tumors, were both markedly inhibited by LQV and ABV. The aforementioned data revealed that in comparison to ABV treatment and DMH control, LQV treatment showed the lowest numbers of ACF criterion (1, 2, 3, ≥ 4). ACFs varied in size; some contained just one aberrant crypt (AC), while others were clustered in foci with two or more aberrant crypts (ACs), three or more (ACs), or four or more (ACs).

**Table 1:** Total ACF number and foci in crypts of colon in CRC-induced and -treated groups

Groups	Total ACF no	No. of aberrant crypt in foci			
		**Foci with 1 crypt**	**Foci with 2 crypts**	**Foci with 3 crypts**	**Foci with ≥ 4 crypts**
**CRC induced**	119.6 ± 36.9	25.9 ± 8.7	24.6 ± 8.4	27.9 ± 9.4	41.2 ± 14.6
**DMH/LQV**	37.4 ± 15.1*	9.9 ± 1.1*	9.4 ± 2.0*	7.6 ± 2.2*	10.2 ± 1.8*
**DMH/ABV**	65.1 ± 14.7*	13.4 ± 2.9*	13.4 ± 4.8*	17.3 ± 5.2*	20.9 ± 5.2*

Means ± SD. *Significant vs. DMH-treated group at *p* ≤ 0.05. *ACF*, aberrant crypt foci; *CRC* induced, CRC induced by DMH; *DMH/LQV*, CRC induced by DMH and treated with LQV; *DMH/ABV*, CRC induced by DMH and treated with ABV

### LQV and ABV treatments significantly decreased MDF in colonic epithelium of CRC-induced groups

**Fig. 4:**
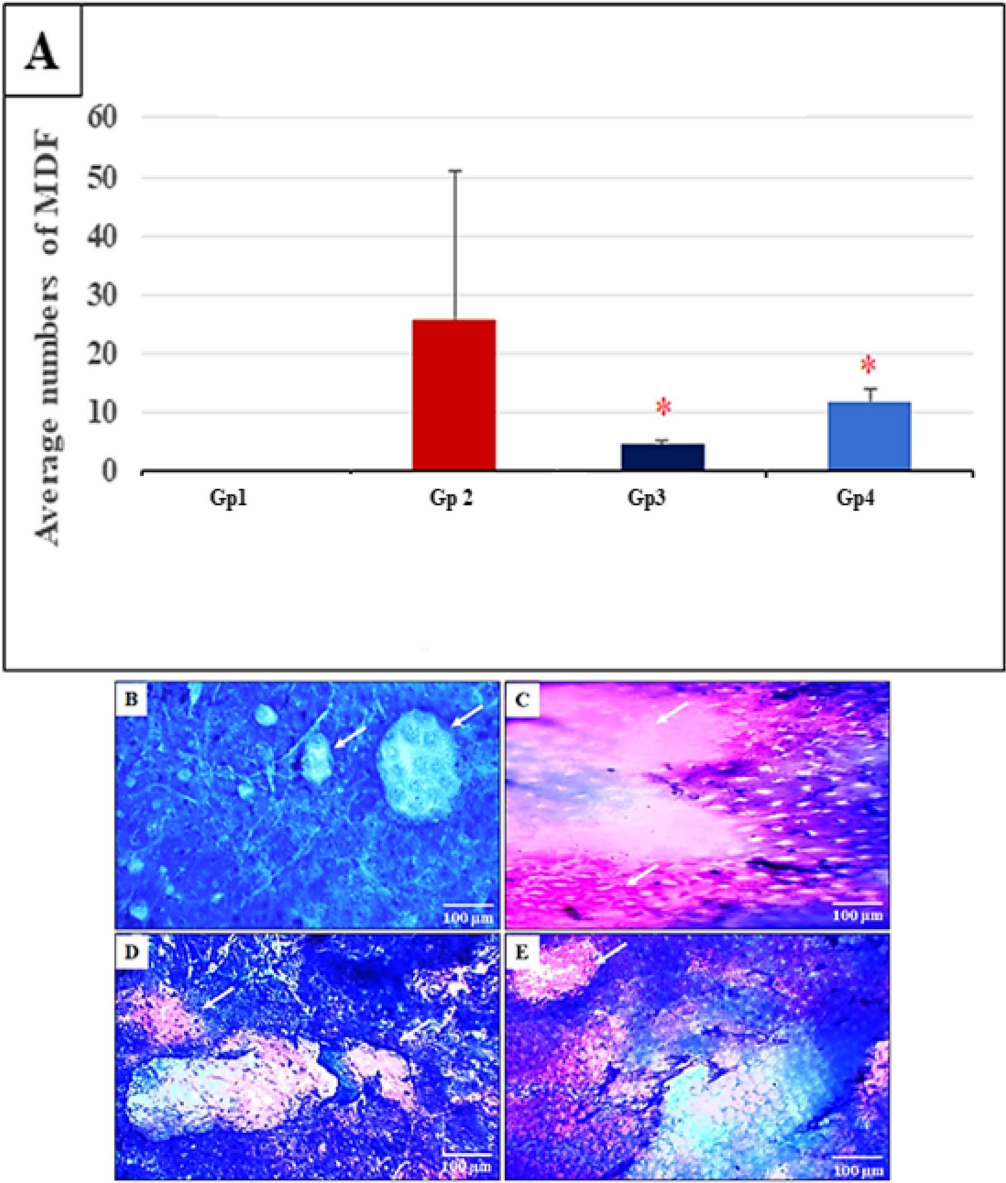
MDF average no. in CRC-induced groups. *Significant vs. G2 at *p* ≤ 0.05. **B**, **C**, **D**, and **E** Showing whole colons stained with Alcian blue-neutral red (AB-NR) which displayed different shapes and sizes of MDF distinguished from the blue-dotted background as a reddish spot in which the crypts do not produce mucin. *t*-test analysis

MDF estimations for each tested rat’s whole colon are displayed; when compared to DMH treated, the rats in DMH/LQV and DMH/ABV treated, respectively, displayed noticeably fewer MDF (*p* < 0.05). Among the DMH-induced groups, it is important to notice that DMH/LQV had the lowest total MDF numbers (Fig. 4).

### The treatment with LQV and ABV increased significantly the goblet cells in colon of CRC-induced groups

**Fig. 5:**
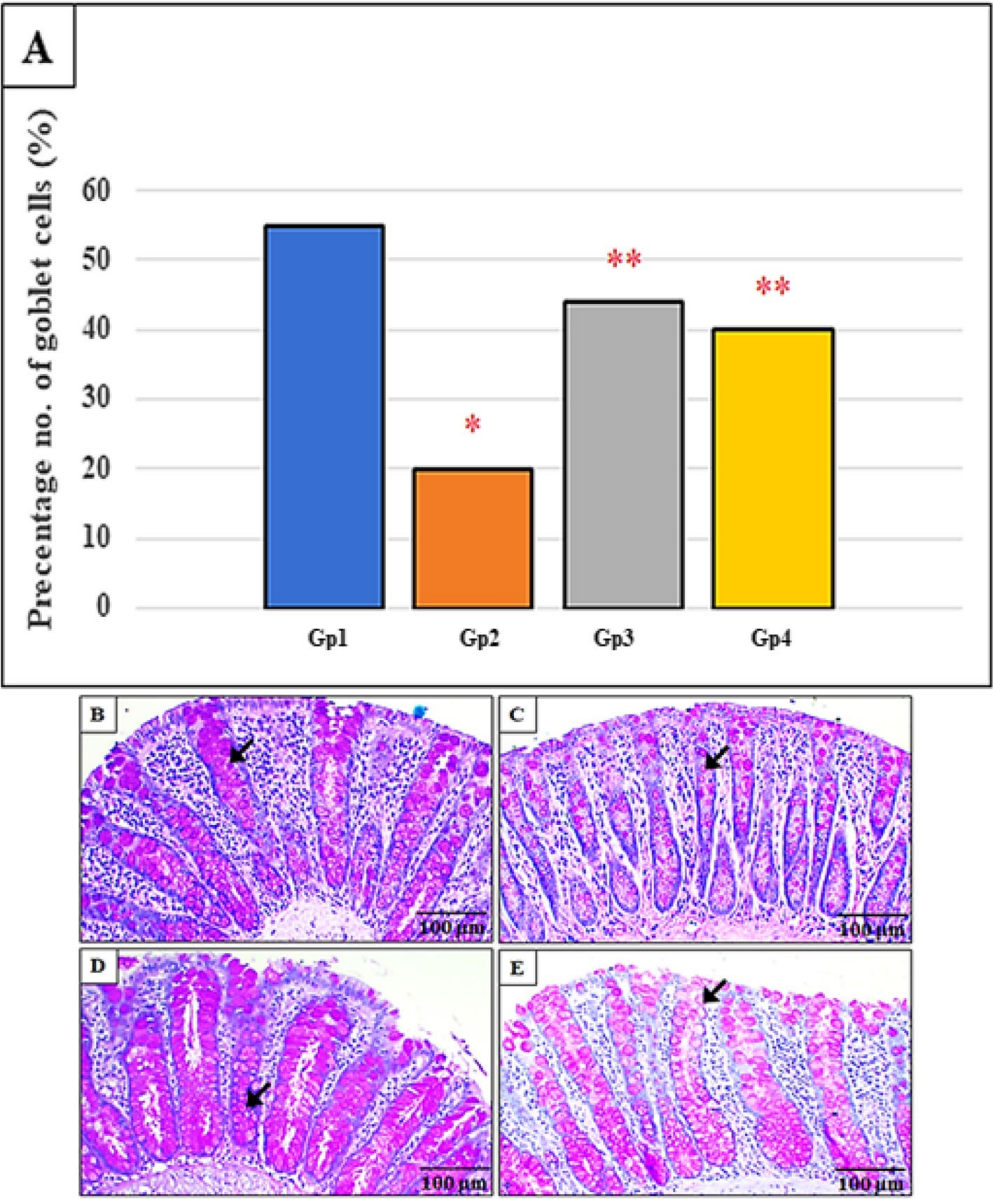
Goblet cells % by PAS histochemical analysis in colon mucosa of control and CRC-induced groups *Significant vs. G1 *p* < *0.05*. **Significant vs. G2 *p* < 0.05. **B** Showing normal colonic epithelium with strong and intense stain of goblet cells (arrow). **C** Colonic epithelium of rat treated with DMH showing faint and weak PAS staining with obviously fewer numbers of goblet cells (arrow). **D** Colonic epithelium of LQV-treated group showed a significant increase in the goblet cell sizes and numbers (arrow). **E** Colonic epithelium of ABV-treated group showing obvious increases in the goblet cell numbers (arrow). Chi-squared analysis

Rats administered DMH showed weak PAS staining of their mucosa and a definite decrease in the number of mucus-secreting cells. The mucosa of the DMH-treated rats receiving LQV and ABV showed a significant increase in goblet cell sizes and numbers as compared to the other groups and similar to the normal control group. Interestingly, ABV or LQV therapy dramatically increased the goblet cells in CRC-induced groups (Fig. 5).

LQV and ABV significantly decreased the expression of PCNA % in colonic crypts of the CRC-induced group.

**Fig. 6:**
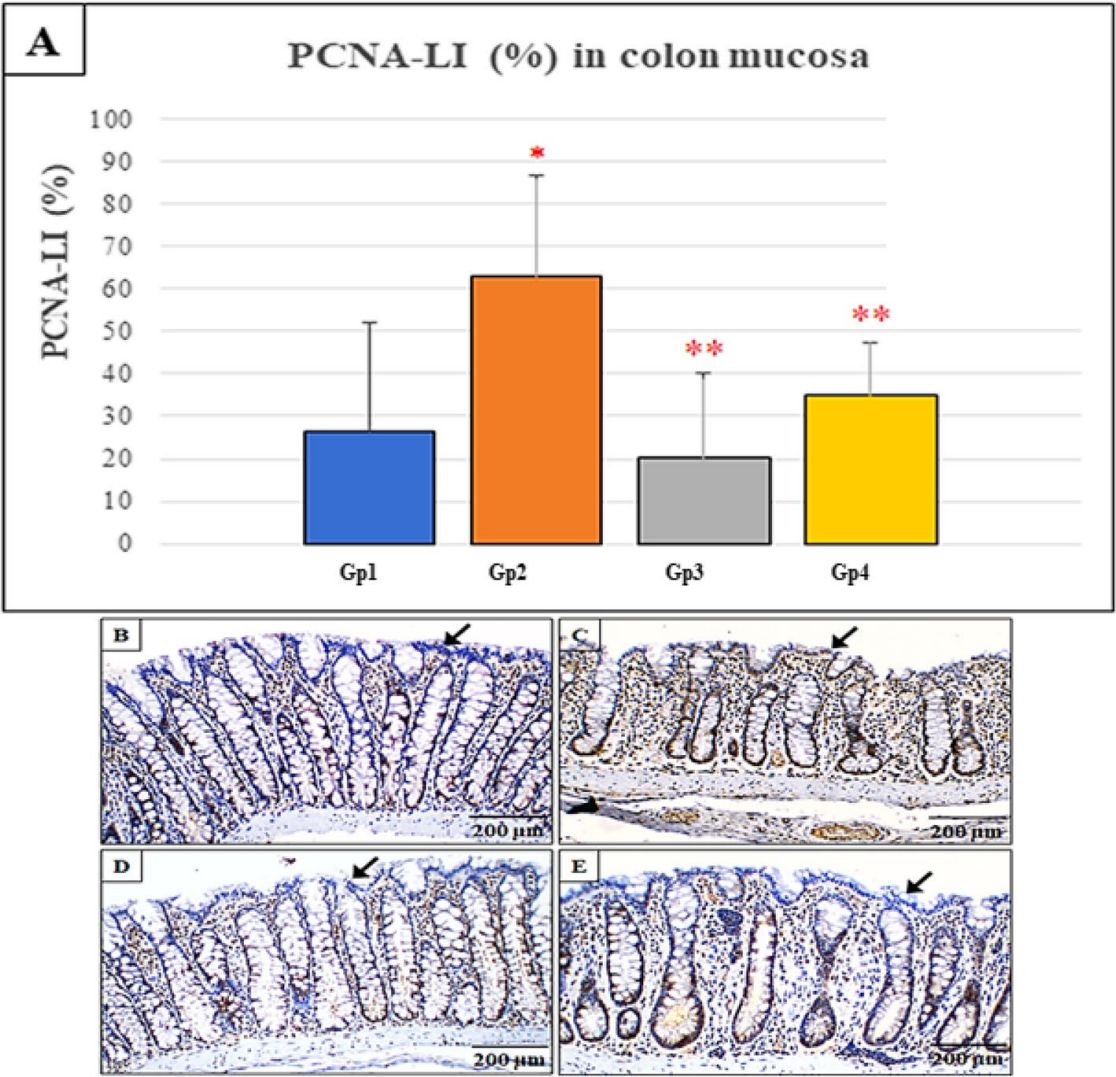
IHC of PCNA-LI (%) showing **A** normally appearing mucosa for all groups and showing a decrease in PCNA-labelled cells after treatment with LQV or ABV. *Significant vs. G1 at *p* < 0.05. **Significant vs. G2 *p* < 0.05. **B** A normal rat’s colonic epithelium with few labelled cells (arrows). **C** Colon of a DMH-only-treated rat showing increased PCNA-labelled cells (arrows). **D** Colonic epithelium showing a significant decrease in PCNA-labelled cells treated with LQV. **E** Colon treated with ABV showed decreased PCNA-labelled cells. *t*-chi-squared analysis

The greatest PCNA LI% was represented in DMH-treated group, compared to the control group. CRC-induced groups treated with LQV and ABV showed a significant decrease in expression in PCNA LI (%) compared to the control groups (Fig. 6).

### PTEN and cyclin-D1 gene expressions

**Fig. 7:**
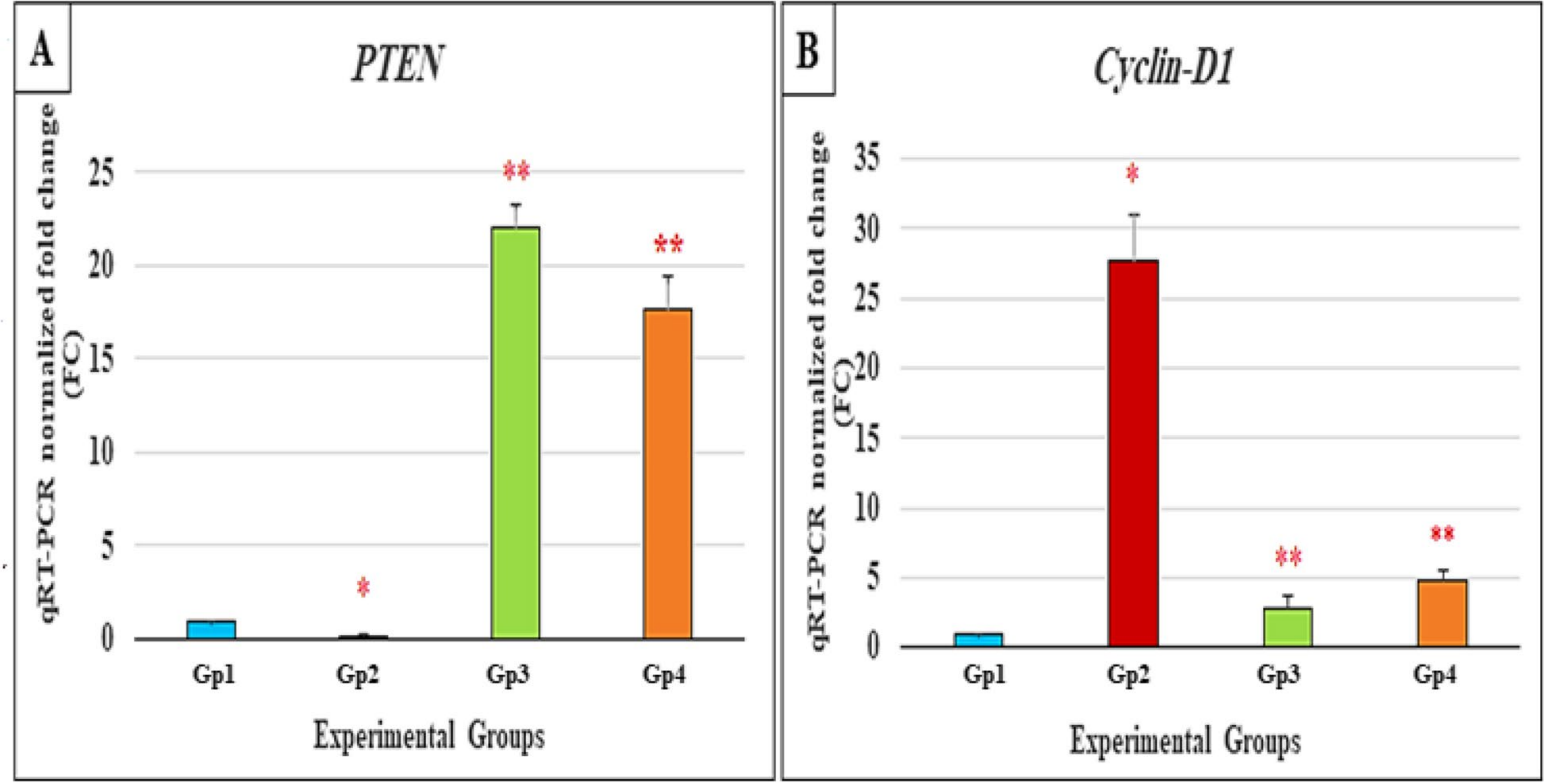
qRT-PCR normalized fold change (FC) data for **A** *PTEN* and **B** *cyclin-D1* genes mRNA expression in the studied groups. Gp1, normal control rats; Gp2, treated with DMH only rats; Gp3, DMH + LQV-treated rats; and Gp4, DMH + ABV-treated rats. *Significant vs. the mRNA expression of all genes studied G1 at *p* < *0.05*. **Significant vs. G2 at *p* < *0.05*. *t*-test analysis

CRC induced chemically by DMH exhibited a significant decrease in the expression of the PTEN gene and increases in the cyclin-D1 gene after 16 wks compared to the control untreated group. The treatment with both LQV and ABV caused an elevation of PTEN gene expression by 22- and 17-fold, respectively, compared to the CRC-induced group, while the expression of cyclin D1 in the LQV-treated group (Gp3) was decreased significantly by 25-fold compared to the CRC-induced group (Gp2). In the ABV-treated group (Gp4), cyclin-D1 expression led to a significant decrease compared to the DMH group (Gp2) by 23-folds (Fig. 7).

### LQV and ABV increase the apoptotic cells% and early and late apoptosis in tumor cells of colon in CRC-induced groups

**Fig. 8:**
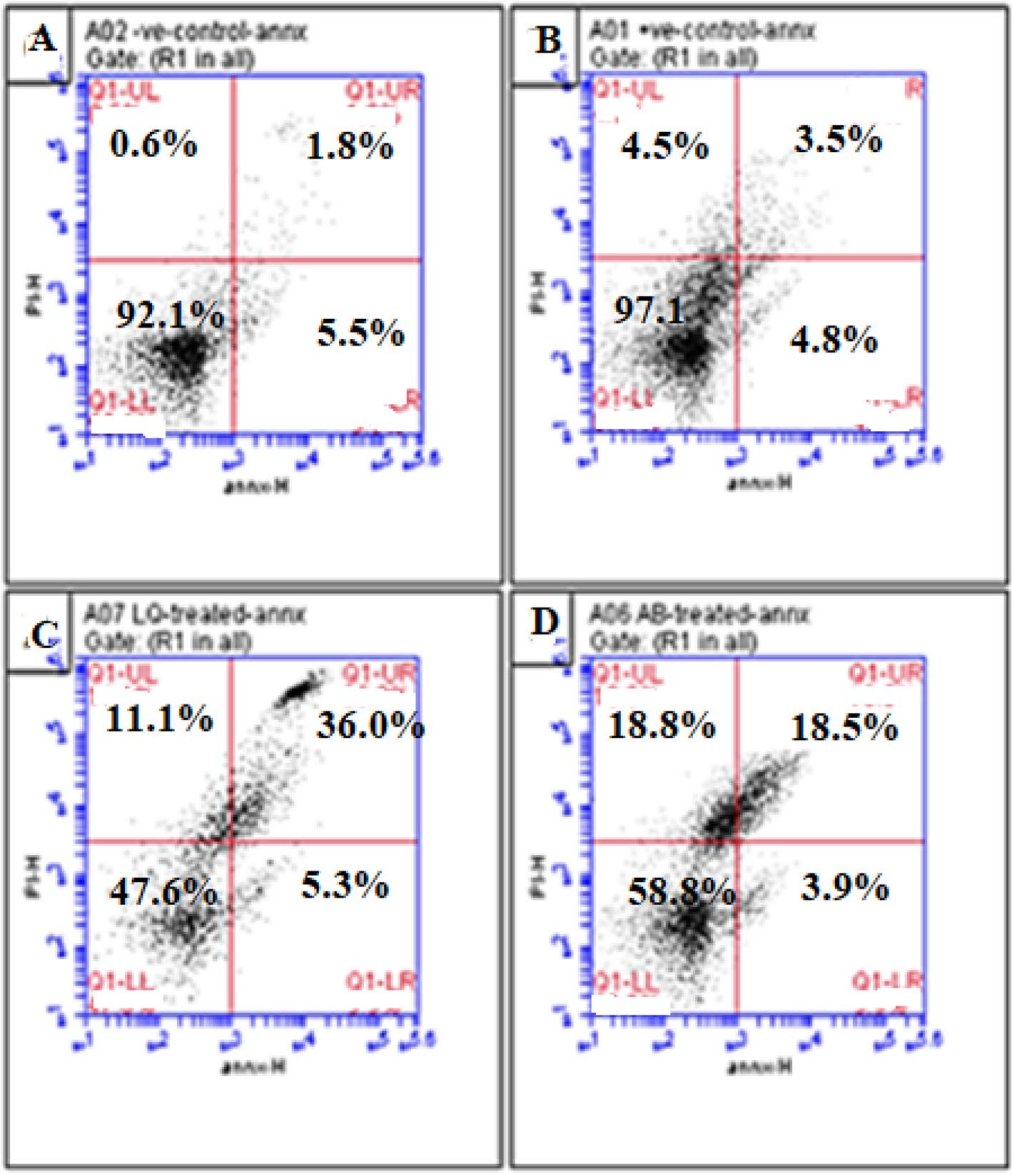
Flow cytometric analysis of control and CRC-induced and -treated groups. **A** Control untreated group. **B** CRC-induced group. **C** CRC-induced group treated with LQV. **D** CRC-induced group treated with ABV

DMH-treated group (Gp2) showed slight increases in apoptotic cells in early apoptosis and significant increases in late apoptosis compared to the control group. LQV- and ABV-treated groups showed significant increases in late apoptosis (36 and 18%, respectively) compared to the DMH group (Fig. 8).

### The treatment with both LQV and ABV showed cell cycle arrest at the sub-G1, G0, and G2/M phases

**Fig. 9:**
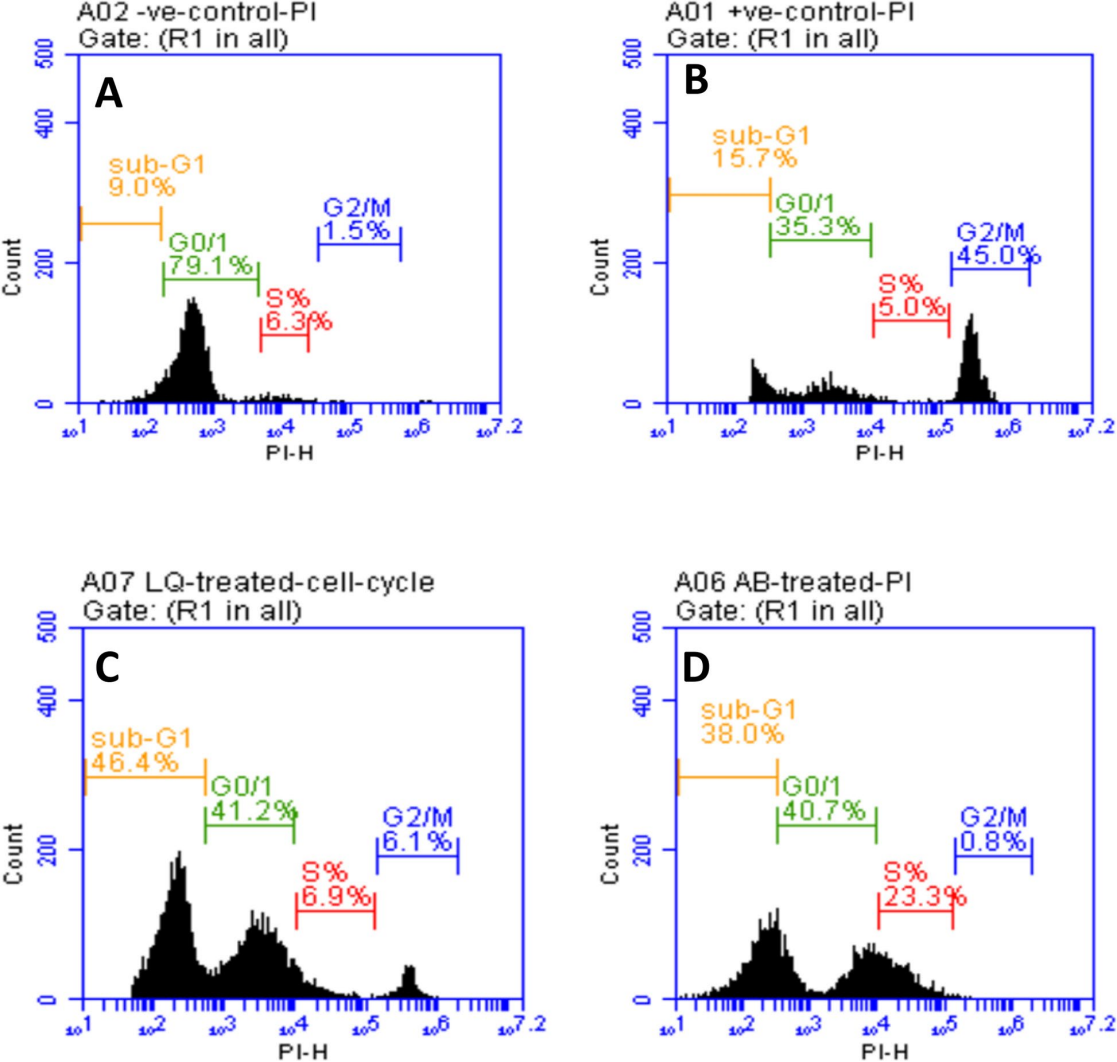
Cell cycle analysis in colon of control and CRC-induced and -treated groups. **A** Control group. **B** CRC-induced group. **C** CRC-induced group treated with LQV. **D** CRC-induced group treated with ABV

The number of cells harvested in the colon of the DMH group was 15.7 ± 2.1, 35.3 ± 5.1, 6.3 ± 0.4, and 45 ± 3.9% at sub-G1, G0/1, S, and G2/M cycle, respectively. Treatment with LQV in the CRC-induced group led to a significant increase in cells harvested in the sub-G1 phase (46.4 ± 1.2%) and in the G0/G1 phase (41.2 ± 1.2%) and decreased the cells harvested in the G2/M phase (6.1 ± 1.6%). Meanwhile, ABV increased the cells harvested in the sub-G1 phase (38 ± 1.1%), in the G0/1 phase (40.7 ± 3.4%), and in the S phase (23.3 ± 4.4.6%). It also decreased the cells harvested in the G2/M phase to 0.8 ± 0.1% (Table 2 and Fig. 9).

**Table 2:** % of cell cycle arrest in each phase of control and CRC-induced and -treated groups

Groups	Sub-G1	G0/G1	S phase	G2/M
Control	9 ± 0.3d	79.1 ± 0.4a	6.3 ± 0.4b	1.5 ± 0.2c
CRC induced	15.7 ± 2.1c	35.3 ± 5.1c	5.0 ± 0.2c	45.0 ± 3.9a
DMH/LQV	46.4 ± 1.2a	41.2 ± 1.2c	6.9 ± 1.5b	6.1 ± 1.6b
DMH/ABV	38.0 ± 1.1b	40.7 ± 3.4b	23.3 ± 4.4a	0.8 ± 0.1d

Values represented as means ± SD. Different letters mean significant *p* < 0.05

## Discussion

Scorpion venoms (SV) have uses in medicine and pharmacology and are composed of proteins, neurotoxins, and lipids [31]. LQV has a cytotoxic effect on several types of cancer cell lines [10] and has anti-tumor activity against Ehrlich ascites carcinoma (EAC)-bearing mice [12]. Also, it has an antidiabetic effect against type 2 diabetes mellitus [32] and ameliorates the lipid profile and hematological parameters in hepatocellular carcinoma induced in rats [33]. Therefore, the present study focused on the potential effect of the venom of two scorpion species as anti-CRC agents.

Colorectal cancer (CRC) is one of the world’s most dangerous public health issues [1]. ACF and MDF are the earliest developing precursors of epithelial neoplasms of the large intestine which appear after 8 to 16 weeks of chemical carcinogen induction. Regarding colon carcinogenesis, they are highly valuable as early indicators [34]. A major focus of research worldwide is creating innovative cancer treatments. Previous investigations have demonstrated that SV contains medicinal substances that may be used as anticancer medications [35]. Compounds found in SV have potential applications in the treatment of cancer [16]. Several investigations, conducted in vitro as well as in vivo, have demonstrated that the crude venom or their peptides, obtained from different species of scorpions, inhibited the development of specific cancer cell types in human pancreatic, colorectal, prostate, glioma, leukemia, lymphoma, lung, breast, liver, and cervix cancer [10, 12, 36].

LQV and ABV may have inhibitory effects on biomarkers ACF and MDF of colorectal cancer, as well as cytotoxicity on CRC cells. This was investigated in the current study using a short-term colon carcinogenicity experiment in rats. The results were in line with earlier studies on the venom of several scorpion species: *Androctonus australis*, *Pandinus cavimanus*, *Scorpio maurus palmatus*, and *Buthus sindicus* which showed cytotoxicity against diverse human cancer cell lines [16, 35, 37, 38].

Based on the current findings, the CRC-induced groups treated with either LQV or ABV showed a significant reduction in the total number of aberrant crypt foci (ACF) compared to the group that received the carcinogen alone. These results align with those of [38, 39], who reported that oral administration of Muricidae extract (derived from a marine gastropod mollusk) suppressed ACF development in a mouse model of colon cancer.

To the best of our knowledge, this is the first study on the effects of SV on colon cancer biomarkers. Additional evidence from natural compounds, such as *Butia capitata* (sour coconut) and marine ascidian *Styela plicata*, has shown similar protective effects by reducing ACF formation in rat models [21, 40]. The current study also found that LQV and ABV significantly decreased MDF numbers, consistent with reports that compounds like aloin (an anthracycline) mitigate DMH-induced damage by lowering ACF and MDF counts [41].

The results of this study demonstrated that treatment with LQV and ABV significantly enhanced the activity of antioxidant enzymes, including SOD, CAT, GPx, and GST, in both liver and colon tissues while reducing the level of MDA, a key marker of lipid peroxidation. This finding suggests a protective role of scorpion venoms against oxidative stress-induced colorectal carcinogenesis. The progression of CRC is closely linked to oxidative damage caused by excessive generation of reactive oxygen species (ROS), which can lead to mutations, genomic instability, and abnormal cell proliferation. Antioxidant enzymes serve as the primary defense mechanism by detoxifying ROS and preventing cellular damage. The observed increase in antioxidant enzyme activities following venom treatment implies a restoration of redox balance and inhibition of oxidative damage, thereby suppressing tumor progression [5].

Goblet cells are specialized for the secretion of mucins, particularly MUC2, which forms the protective mucus layer lining the colon epithelium [41]. An increased mucin layer serves as a physical and biochemical barrier, shielding epithelial cells from direct exposure to carcinogens, bile acids, and pro-inflammatory cytokines. This barrier reduces epithelial irritation, inflammatory cell infiltration, and ultimately the risk of neoplastic transformation. Mucins secreted by goblet cells have been shown to modulate immune responses, reduce oxidative stress, and suppress pro-tumorigenic signaling pathways. In preneoplastic lesions, goblet cell depletion is often an early sign of dysplasia or carcinoma in situ. Conversely, preserved or enhanced goblet cell differentiation indicates that normal mucosal architecture is being maintained [42]. Histochemical analysis showed that DMH-treated rats had fewer mucin-secreting goblet cells, with weak PAS staining. However, rats treated with LQV and ABV displayed significantly increased goblet cell numbers and improved PAS staining, indicating enhanced mucosal protection. This aligns with a previous study in which bee venom improved carbohydrate levels and protected against liver and kidney toxicity in PAS-stained tissues [42]. Bee venom was also shown to mitigate LPS-induced acute kidney injury [43].

Unregulated cell proliferation is a hallmark of cancer, and malignant cell proliferation is the fundamental cause of cancer; cell proliferation is an essential property of all living things. The cell proliferation marker protein, PCNA, was substantially elevated in the group that received only DMH; this indicates unregulated cell proliferation and is related to ACF multiplicity in the colon mucosa [44]. The current work findings showed a considerable reduction in the number of PCNA-labelled cells in the colon cancer mucosa following treatment with either LQV or ABV. According to [38], PCNA expression in solid Ehrlich tumor tissues was found to be considerably reduced by the Smp24 peptide, which is extracted from *S. maurus palmatus* venom. Additionally, previous findings indicate that treatment with additional natural compounds, such as eupafolin, greatly reduced the number of PCNA-labelled cells, hence raising the cell death rate [44]. Recent research has reported that *TP53* regulates PCNA levels to govern DNA replication. Therefore, the present findings suggest that either ABV or LQV treatment elevated the *TP53* level, which in turn boosted the apoptotic process of the cancer cells and restricted their growth, thereby decreasing the number of ACFs and hyperplastic lesions [45, 46]. The ratio of early apoptotic to late apoptotic/necrotic cells was greater, according to our flow cytometric examination of apoptosis.

The carcinogen, DMH, caused slight elevation in early and late apoptosis in the current study. While LQV and ABV significantly increased the late apoptosis compared to the DMH-treated group. This data was compatible with the study of [10] who stated that LQV increased % apoptotic cells in early and late apoptosis of colorectal cells. Furthermore, LQV increased the number of necrotic and apoptotic cells in the EAC-treated group [12]. Earlier research has shown that components of SV and flow cytometry assays can be used to assess apoptosis in cancer cell lines at an early stage [47, 48]. According to [49], *Heterometrus bengalensis*, the Indian black scorpion, can significantly increase the amount of apoptosis that occurs in leukemic U937 and K562 cells when exposed to Koch crude venom. Based on the research of [50], *Hemiscorpius lepturus* venom can profoundly alter highly specialized CT26 cells, raising the apoptotic ratio above 60%.

Furthermore, as demonstrated by the cell cycle analysis using flow cytometry, which examined the apoptotic cell rate, this study found the ability of LQV and ABV to stop proliferation and division of colon cancer cells. Between the DMH-only group and the LQV- and ABV-treated groups, it rose from 15.0% to 46.1% and 40.2%, respectively. Consequently, the cell cycle phase data from LQV and ABV demonstrated that they both cause cell cycle arrest at G2/M and were accompanied by a significantly enhanced S phase in the ABV and a somewhat elevated S phase in the LQV when compared to the DMH group. LQV arrested the cell cycle at G0 and S phases in EAC-bearing mice [11]. The present data is consistent with the assessment made by [51] which showed that the Smp24 peptide, extracted from the venom of the *S. maurus palmatus*, markedly elevated the rate of apoptosis in HepG2 cells, rising from 8.64% to 46.8%. Furthermore, it causes the S and G2/M phases of the HepG2 cell cycle to be stopped. A crucial method by which cancer therapies interact with the body is via the strictly regulated process of apoptosis [52]. It is commonly known that mitochondrial damage has a role in the accumulation of the cell cycle, which leads to the death of cancer cells [53]. Interstingly, scorpion venoms have been capable of stopping the cell cycle at multiple phases as; G0/G1, G2/M, and G1/S [54].

To determine the molecular mechanism underlying G2/M cell cycle arrest induced by LQV or ABV, more research was done on the expression of cell cycle regulating proteins such as *PTEN* and *cyclin D1. Cyclin D1* is one of the members of the cyclin family that regulates the transition of cells from the G1 to the S phase [55]. The development of the G2/M transition is primarily regulated by the cyclin-dependent kinase 1 (CDK1)/cyclin B complex. Strong cyclin-dependent kinase inhibitor p21^Waft1/Clip1^ is one significant regulator that inhibits the G2/M phase and reduces CDK1 activity. As per [56], this contributes to the explanation of the inhibition of cancer cell development. The results of this study indicate that LQV or ABV inhibited *cyclin D1*, causing cells to stop G2/M checkpoints and a strong G2/M cell cycle arrest in colon cancer cells. A previous study reported that LQV downregulated the anti-apoptotic gene (bcl-2) and upregulated apoptotic-related genes (Bax and caspase-3) expressions in EAC cells [12]. Smp24 peptide was extracted from the venom of *S. maurus palmatus* and tested on the hepatoma cell line HepG2, as reported by [51]. As per the findings, Smp24 caused cell cycle accumulation at the S and G2/M phases in HepG2 cells by suppressing the production of *cyclin E* and CDK2 while upregulating the expression of *cyclin A*, *cyclin-B*, p21^Waft1/Clip1^, and p53. The impact of administering snake venom *Echis coloratus* and *Walterinesia aegyptia* on colon cancer cells, HT-29 and LoVo cultures, and snake venom inhibited the growth of colon cancer cells by inhibiting *cyclin D1* [57]. BmK peptides inhibited the growth of cultured human hepatoma (SMMC7721) and breast cancer (MCF-7) cells by upregulating caspase-3, causing apoptosis, stopping the cell cycle from entering the S phase and going from the G0/G1 phase to the G0/G1 phase, and reducing the amounts of the cell cycle regulation-related protein *cyclin D1* [58]. The expression of cell cycle-regulating proteins such as *PTEN* was also examined. *PTEN* is the tumor suppressor gene that negatively regulates PI3K. Moreover, the PI3K/Akt/mTOR pathways are considered potential targets for cancer therapy due to their importance in the processes of cell growth, proliferation, survival, and motility [59]. Our findings demonstrated that the PTEN/PI3K/Akt/mTOR pathway inhibited cell cycle progression and the G2/M transition in colon cancer mucosa following treatment with LQV and ABV by increasing *PTEN* levels. This was in line with a study by [60] that showed gonearrestide inhibits the growth of primary colon cancer cells and solid tumors by causing cell cycle arrest in the G1 phase through increased *PTEN* levels and inhibition of the Akt pathway. This was in line with a study by [61] that found that treating human colon cancer SW480 cells with scorpion venom elevated *PTEN*.

Furthermore, this elevation resulted in decreased PI3K/Akt signal transduction, which caused SW480 cells to enter the G0/G1 cell cycle arrest. These findings, which were obtained using portions of colon tissue, suggest that scorpion venom plays a proactive role in inducing apoptosis and cellular proliferation. Meanwhile, the generation of extremely dangerous hydroxyl radicals is suppressed by antioxidant enzymes [62]. Our findings demonstrated that in comparison to the DMH group, the administration of LQV and ABV significantly increased the activity of antioxidant enzymes in the liver and colon mucosa. This is consistent with studies [35, 38, 63] that demonstrate elevated levels of antioxidant enzymes and lipid peroxidation following venom therapy. Interestingly, some types of scorpion venom are rich in chlorotoxin-like peptides, which selectively bind to matrix metalloproteinases (MMPs) and glioma-specific chloride channels. These properties have been linked to anti-invasive and anti-metastatic activities and contain potent neurotoxins that modulate Na⁺, K⁺, and Cl − ion channels, which are increasingly recognized as regulators of cancer cell proliferation and apoptosis, and other peptides have been shown to impair angiogenesis and tumor cell motility. Also, it has dual mechanisms: direct pro-apoptotic effect through ROS induction and mitochondrial disruption in tumor cells and indirect chemopreventive effect through modulation of antioxidant enzymes and immune response, reducing the tumor-promoting microenvironment [64–66].

## Conclusion

LQV and ABV demonstrated potent anticancer effects against colon cancer in vivo. They induced tumor cell apoptosis, inhibited proliferation, and enhanced antioxidant defenses by raising antioxidant enzyme levels and reducing oxidative stress. These venoms also blocked ion channels in malignant cells, suppressing key pathways like PI3K/Akt/mTOR and causing cell cycle arrest in G1, G2, and S phases, thereby preventing uncontrolled cancer cell growth. Finally, LQV and ABV show promise as apoptotic and anti-colorectal cancer therapy with reduced toxicity and side effects when compared to other pharmaceuticals and chemotherapeutic drugs.

### Limitations

While the current study demonstrates promising anticancer potential of *L. quinquestriatus* venom (LQV) and *A. bicolor* venom (ABV) in a chemically induced rat model of colorectal cancer, several limitations must be acknowledged:

- *Short-term assessment*: The experimental duration (16 weeks) provides insight into early and intermediate events in carcinogenesis and response to treatment. However, long-term outcomes such as tumor recurrence, metastasis, and survival were not evaluated.
- *Crude venom use*: The study utilized crude scorpion venom, which contains a mixture of bioactive compounds. Isolation, identification, and mechanistic assessment of the individual active peptides or molecules are necessary to clarify their specific roles and enhance reproducibility.
- *Limited molecular targets*: While key biomarkers like PCNA, cyclin D1, and PTEN were evaluated, broader transcriptomic or proteomic analyses would provide a more comprehensive understanding of the pathways modulated by SV treatment.
- *Translation to clinical use*: The pharmacokinetics, immunogenicity, and formulation stability of scorpion venom peptides remain uncharacterized, which are critical factors for drug development and human application.

## Data Availability

No datasets were generated or analysed during the current study.

## Abbreviations

ABV: *Androctonus bicolor* Venom
ACF: Aberrant crypt foci
CAT: Catalase
CRC: Colorectal cancer
DMH: 1,2-Dimethylhydrazine
GPX: Cellular glutathione peroxidase
GST: Glutathione S-transferase
IHC: Immunohistochemistry
LD50: Median lethal dose
LQV: *Leiurus quinquestriatus* Venom
MDA: Malondialdehyde
MDF: Mucin-depleted foci
SD: Standard deviation
SOD: Superoxide dismutase
SV: Scorpion venom
